# Association between multidrug resistance-1 C3435T gene polymorphism and right ventricular dysfunction in patients with chronic obstructive pulmonary disease: cross-sectional study

**DOI:** 10.1590/1516-3180.2017.0299281017

**Published:** 2018-01-09

**Authors:** Oğuzhan Yücel, Hakan Güneş, Hasan Yücel, Ali Zorlu

**Affiliations:** I MD. Physician, Department of Cardiology, Anatolian Hospital Samsun, Turkey; II MD. Assistant Professor, Department of Cardiology, Kahramanmaraş Sütçü İmam Üniversitesi, Kahramanmaraş, Turkey; III MD. Associate Professor, Department of Cardiology, Cumhuriyet Üniversitesi Tıp Fakültesi, Sivas, Turkey; IV MD. Associate Professor, Department of Cardiology, Cumhuriyet Üniversitesi Tıp Fakültesi, Sivas, Turkey

**Keywords:** Pulmonary disease, chronic obstructive, Polymorphism, genetic, Ventricular dysfunction, right, Circulation, Pulmonary

## Abstract

**BACKGROUND:**

Right ventricular (RV) dysfunction may develop over the course of chronic obstructive pulmonary disease (COPD) and is an important predictor of morbidity and mortality. Polymorphism of the multidrug resistance-1 (MDR-1) gene has been correlated with worse clinical findings among patients with COPD. Our aim here was to investigate the relationship between MDR-1 C3435T gene polymorphism and RV dysfunction in COPD patients.

**DESIGN AND SETTING:**

This was a cross-sectional study investigating the relationship between RV dysfunction and genetic defects in COPD patients.

**METHODS:**

Forty-one consecutive patients diagnosed with COPD and hospitalized due to acute exacerbation were enrolled. Polymorphism was analyzed using the strip assay technique. RV parameters were evaluated, and RV dysfunction was identified via transthoracic echocardiography. Patients were categorized into three groups according to gene polymorphism: MDR-1 CC (wild type, n = 9), MDR-1 CT (heterozygote mutant, n = 21) or MDR-1 TT (homozygote mutant, n = 11).

**RESULTS:**

The study included 14 males and 27 females (mean age 65 ± 11 years). The mean systolic pulmonary artery pressure was 31.4 ± 8 mmHg in the wild-type group, 42.2 ± 12 mmHg in the heterozygote mutant group and 46.5±14 mmHg in the homozygote mutant group (P = 0.027). Presence of RV dilatation was significantly different among the three groups (33%, 71%, and 100%, respectively; P = 0.005). In multiple logistic regression analysis, MDR-1 C3435T gene polymorphism (OR = 9.000, P = 0.019) was an independent predictor of RV dysfunction after adjustment for potential confounders.

**CONCLUSION:**

MDR-1 C3435T gene polymorphism was associated with RV dysfunction in patients with COPD.

## INTRODUCTION

Chronic obstructive pulmonary disease (COPD) gives rise to important morbidity and mortality over its progressive course. Pulmonary hypertension (PHT), right ventricular (RV) failure and cor pulmonale may develop over this course, and these are important predictors of morbidity and mortality in COPD. There is a growing volume of data on the roles of pulmonary and systemic inflammation and genetic factors in the onset and progression of COPD.^1^^,^^2^^,^^3^^,^^4^

The multidrug resistance-1 (MDR-1) gene, which is responsible for drug resistance, has a role in transportation of ions and peptides and in elimination of toxic substances.^5^ It has been suggested that the multidrug resistance associated protein-1 (MRP-1), which is a product of this gene, has a role in antioxidative metabolism in the lungs. MRP-1 is expressed to a lesser extent in the bronchial epithelium of COPD patients than in that of healthy subjects.^6^ MRP-1 levels have ben correlated with the severity of COPD.^7^ Furthermore, single nucleotide C3435T gene polymorphism of the MDR-1 gene is associated with reduced MRP-1 levels.^8^

The aim of the present study was to investigate the impact of this gene polymorphism on RV dysfunction in patients with COPD.

## METHODS

This study was performed in accordance with the Declaration of Helsinki for Human Research, and was approved by Cumhuriyet University Institutional Review Board (protocol number 2010/70, June 2010).

Forty-one consecutive patients who had previously been diagnosed with COPD and had been hospitalized due to acute exacerbation were enrolled into the study. Patients with other cardio-pulmonary diseases were excluded. After informed consent had been obtained, a 5-ml peripheral blood sample was taken from each participant for genetic analysis. The patients were categorized into three groups according to this genetic analysis: MDR-1 CC (wild type), MDR-1 CT (heterozygote mutant) or MDR-1 TT (homozygote mutant).

Total genomic DNA was extracted from 100-ml blood samples using the Invitek kit (Invisorb spin blood, Invitek, Germany). The MDR-1 gene was amplified in a biotin-labeled single multiplex amplification reaction and was evaluated for the 3435 C > T polymorphism. The polymerase chain reaction (PCR) was performed in a Perkin-Elmer Geneamp 9600 thermal cycler. The protocol consisted of an initial melting step of 2 minutes at 94 °C, followed by 35 cycles of 15 seconds at 94 °C, 30 seconds at 58 °C and 30 seconds at 72 °C, and a final elongation step of 3 minutes at 72 °C. Polymorphism analysis was performed using the strip assay technique (ViennaLab, PGX-HIV Strip Assay GmbH, Austria), which is based on reverse hybridization.

Echocardiographic examinations were performed via the Vivid 7 system (GE Healthcare, Wauwatosa, WI, USA) with 2.5 to 5-MHz probes. The ejection fraction was calculated by means of the modified Simpson method. Chamber sizes were defined in accordance with recent guidelines.^9^ In order to evaluate right ventricular (RV) dysfunction, the presence of RV dilatation, increased tricuspid regurgitation jet flow rate and increased systolic pulmonary artery pressure (sPAP) were evaluated on echocardiography. RV dimensions were evaluated in accordance with the most recent guideline,^9^ and RV dimension > 3.4 cm in the basal plane or > 3.8 cm at mid-plane was used to designate RV dilatation as per the guidelines. Right atrium size was measured across the minor-axis dimension, extending from the lateral border of the right atrium to the inter-atrial septum.^9^ Valvular regurgitations were graded into categories (trivial, mild, moderate or severe) via combinations of Doppler jet color flow signal intensity and vena contracta width, in accordance with the guideline recommendations.^10^ Systolic pulmonary artery pressure was calculated as shown previously.^11^ Echocardiography was performed twice by two experienced cardiologists who were blind to the patients’ genotype.

Hypertension was deemed to be present in situations of blood pressure > 140/90 mmHg on more than two occasions during office measurements or being on antihypertensive treatment. Diabetes mellitus was deemed to be present in situations of fasting blood glucose ≥ 126 mg/dl or being on antidiabetic treatment. Individuals who continued smoking during index admission were considered to be current smokers. Heart rate and laboratory findings such as C-reactive protein levels, sedimentation rate and arterial blood gas levels were evaluated. The study was performed in accordance with the Declaration of Helsinki for Human Research, and was approved by our institutional review board.

Parametric data were expressed as mean ± standard deviation, and categorical data as percentages. The Statistical Package for the Social Sciences 15.0 (SPSS, Inc., Chicago, Illinois, USA) was used to perform statistical procedures. Comparisons between groups were performed by using one-way analysis of variance (ANOVA) with post-hoc analysis by means of Tukey’s honest significant difference (HSD) test or an independent-sample t test. The Kruskal-Wallis test or the Mann-Whitney U test was used for normally or abnormally distributed data, respectively. Categorical data were evaluated by means of the chi-square test, as appropriate. Multivariable logistic regression analysis was used to evaluate independent clinical parameters that predicted RV dysfunction. A P-value of 0.05 was considered significant.

## RESULTS

The study included 14 males and 27 females, with a mean age of 65 ± 11 years. The baseline characteristics of the patients with COPD, classified into three categories according to their MDR-1 C3435T gene polymorphism, are presented in **Table 1**. The baseline characteristics, laboratory findings and echocardiography parameters (except for sPAP and RV dilatation) did not differ among the three groups (P > 0.05).

**Table 1.** Baseline characteristics of study patients

	Wild type (n = 9)	Heterozygote mutants (n = 21)	Homozygote mutants (n = 11)	P
**Baseline characteristics**				
Age (years)	66 ± 11	64 ± 13	67 ± 10	0.760
Gender (male/female)	5/4	6/15	3/8	0.308
Hypertension	6 (67%)	15 (71%)	8 (73%)	0.952
Diabetes mellitus	1 (11%)	5 (24%)	5 (46%)	0.205
Smoking	4 (44%)	5 (24%)	2 (18%)	0.379
Atrial fibrillation	2 (22%)	10 (48%)	6 (55%)	0.310
**Echocardiography parameters**				
Ejection fraction (%)	60 ± 3	59 ± 5	59 ± 4	0.751
Left ventricular diastolic dysfunction	7 (78%)	17 (81%)	11 (100%)	0.269
Mitral regurgitation (trivial/mild/moderate/severe)	2/7/0/0	7/11/3/0	2/9/0/0	0.312
Aortic regurgitation (trivial/mild/moderate/severe)	7/2/0/0	14/5/2/0	8/3/0/0	0.721
Left atrium size (cm)	4.2 ± 1.3	4.1 ± 0.6	4.4 ± 0.5	0.632
Tricuspid regurgitation (trivial/mild/moderate/severe)	1/5/3/0	0/11/7/3	0/6/4/1	0.555
Systolic pulmonary artery pressure (mmHg)	31.4 ± 8.4	42.2 ± 12.3	46.5 ± 14.3	0.027
Right ventricular dilatation	3 (33%)	15 (71%)	11 (100%)	0.005
**Laboratory parameters**				
C-reactive protein (mg/l)	14 ± 11	29 ± 30	20 ± 28	0.463
Sedimentation (mm/s)	12 ± 12	15 ± 18	16 ± 20	0.862
Arterial pH	7.42 ± 0.05	7.41 ± 0.04	7.44 ± 0.05	0.301
pO_2_ (torr)	57 ± 10	55 ± 19	59 ± 15	0.811
pCO_2_ (torr)	43 ± 9	42 ± 8	44 ± 8	0.814
Oxygen saturation (%)	88 ± 11	86 ± 9	88 ± 8	0.881

**Figure 1.**
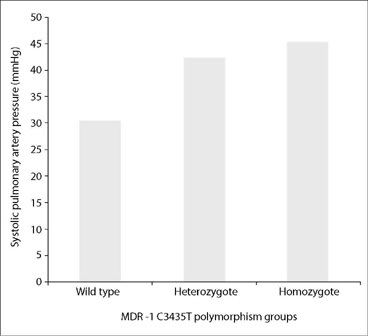
Comparison of systolic pulmonary artery pressure (sPAP) levels between the groups.

The mean sPAP was 31.4 ± 8 mmHg in the wild-type (CC) group, 42.2 ± 12 mmHg in the heterozygote mutant (CT) group and 46.5 ± 14 mmHg in the homozygote mutant (TT) group (P = 0.027; **Figure 1**). The presence of RV dilatation was significantly different among the three groups (33%, 71%, and 100%, respectively, P = 0.005, **Table 1**). In the multivariate logistic regression analysis, MDR-1 C3435T gene polymorphism (odds ratio = 9.000; 95% confidence interval = 1.446-56.022; P = 0.019) was found to be associated with RV dysfunction after adjustment for potential confounders (age, gender, oxygen saturation, presence of hypertension, diabetes mellitus, smoking and atrial fibrillation).

## DISCUSSION

The findings from this study demonstrated that C3435T polymorphism of MDR-1 gene was associated with RV dysfunction in patients with COPD.

Right ventricular dysfunction is associated with shorter survival and frequent episodes of exacerbation in cases of COPD.^12^^,^^13^ Hypoxic vasoconstriction, mechanical stress on hyperinflated lungs, loss of capillaries, inflammation and toxic effects from cigarette smoke are the pathophysiological mechanisms for pulmonary hypertension (PHT) and also for RV dysfunction in COPD.^11^

In COPD, PHT is more prevalent at advanced stages, but it is generally moderate, with mean sPAP < 50 mmHg. The patients in the present study were at stage 2 or 3 and their mean sPAP was 41.0 ± 13 mmHg. The rate of cigarette smoking was 27% among all the patients and it did not differ between the groups. The underlying pathophysiological mechanisms for elevation of sPAP can be considered to have been similar between all the groups. The differences in RV dysfunction among the three groups can be explained in terms of the confounding effects of C3435T polymorphism in this process, caused especially by excessive inflammation.

We previously reported that there was a high frequency of C3435T polymorphism of the MDR-1 gene in patients with COPD, compared with healthy controls.^14^ The association between the MDR-1 gene and the presence and severity of COPD has also been clearly described.^4^^,^^15^ This association was attributed to the roles of the MDR-1 gene and MRP-1 in the antioxidant system and inflammation process. MRP-1 plays an important role in normal lung physiology through protecting against toxic xenobiotics and endogenous metabolites.^5^ Cigarette smoke extracts inhibit MRP-1 activity in bronchial epithelial cells in vitro.^16^ MRP-1 plays a role in combating the toxic effects of smoking and in the removal of oxidative stress metabolites.^17^^,^^18^ The MDR-1 gene also plays a role in cell regeneration.^19^ Pro-inflammatory cytokines decrease the amount of products (MRP-1) secreted from cells.^20^ A decrease in MRP-1 expression and activity has been observed during inflammation.^21^ Lower levels of MRP-1 due to MDR-1 C3435T polymorphism, as previously noted, could be the cause of excessive inflammation and thus a higher proportion of RV dysfunction.

## CONCLUSION

It is not known which patients with COPD will develop RV dysfunction, even though the factors contributing to the development of RV dysfunction are known. This study showed that patients with COPD who carry the mutant allele for the MDR-1 gene are at high risk of development of RV dysfunction. Future studies with larger groups may reveal whether these genetic alterations have any significant impact on RV dysfunction or not.
